# Repetitive transcranial magnetic stimulation for psychiatric symptoms in long-term hospitalized veterans with schizophrenia: A randomized double-blind controlled trial

**DOI:** 10.3389/fpsyt.2022.873057

**Published:** 2022-09-23

**Authors:** Xiuru Su, Long Zhao, Yujie Shang, Yingnan Chen, Xiaowen Liu, Xuan Wang, Meihong Xiu, Huijing Yu, Lijun Liu

**Affiliations:** ^1^Hebei Province Veterans Hospital, Baoding, China; ^2^Peking University HuiLongGuan Clinical Medical School, Beijing HuiLongGuan Hospital, Beijing, China

**Keywords:** schizophrenia, rTMS, randomized controlled trial, clinical outcome, veteran

## Abstract

Multiple lines of evidence demonstrate that high-frequency (HF) repetitive transcranial magnetic stimulation (rTMS) may improve clinical outcomes in patients with schizophrenia (SCZ). However, the efficacy of HF-rTMS on psychiatric symptoms remains unknown in veterans with SCZ. This study aimed to investigate whether HF-rTMS was beneficial in alleviating the clinical symptoms in veterans with SCZ. Forty-seven long-term hospitalized veterans with SCZ were randomly allocated to receive neuronavigated 10 Hz rTMS or sham stimulation over the left dorsolateral prefrontal cortex once daily for four consecutive weeks. Symptoms were assessed by using the Positive and Negative Syndrome Scale at baseline and at the end of week 4. We also collected easily available routine biochemical markers including blood sugar, lipid profiles, hormone, and blood cell counts, considering that these markers may potentially be used to predict the outcomes of rTMS treatment. We found that there was a significant interaction effect of time and group on the positive symptoms. Compared with the sham group, the positive factor score of veterans with SCZ was significantly decreased after treatment in the real rTMS group. Interestingly, the improvement of positive symptoms from baseline to 4-week follow-up was significantly associated with the whole white blood cells (WBC) counts at baseline in the real rTMS group, and baseline WBC counts were predictive of the symptom improvement after rTMS treatment. Our findings indicate that add-on 10 Hz rTMS is beneficial for clinical symptoms in veterans with SCZ. In addition, the baseline WBC counts were predictive of the outcomes after treatment.

**Clinical trial registration:**

clinicaltrials.gov, identifier NCT03774927.

## Introduction

Schizophrenia (SCZ) is a chronic brain disease characterized by disordered thoughts, abnormal behaviors and social interactions, and impaired cognitive functions (1). It is estimated that in the United States, the prevalence of SCZ among veterans is approximately 3% (2). In China, veterans with SCZ need to be professionally treated and cared for in special psychiatric hospitals, and the Office of Veterans Affairs is facing a growing demand for long-term care and a large economic burden (3, 4).

Antipsychotics, such as oral antipsychotic therapy and long-acting injection, are the most commonly prescribed medications for patients with SCZ (5, 6). Although long-lasting, continuous antipsychotic medication is pivotal for patients with SCZ, treatment adherence is often difficult for patients (4, 7). Moreover, a high rate of relapse is observed among SCZ patients who have been receiving antipsychotic medication. Therefore, a majority of patients with SCZ experience multiple relapses and rehospitalization during the course of the disorder (8, 9). Studies reported that the readmission rate of patients with SCZ was 70.5% within 10 years, and the readmission rate was 25% within 4 months after the first hospitalization (10, 11). In addition to the high relapse after treatment, the efficacy of antipsychotics is also a challenge. According to the CATIE trial that reported an all-cause discontinuation combined with efficacy and tolerance, more patients discontinued due to its ineffectiveness (40%) than side effects (20%) (12).

Repetitive transcranial magnetic stimulation (rTMS) is a safe and non-invasive treatment technique used to treat various psychiatric disorders. It has been shown to be a therapy option that is beneficial for patients with SCZ (13, 14). rTMS is favorably safe without systemic adverse events (e.g., tardive dyskinesia, weight gain, and diabetes). The most widely accepted mechanism for the neurological effects of rTMS is that rTMS can alter synaptic plasticity, primarily the long-term enhancement/inhibition of excitatory synaptic transmission (LTP/LTD) (15). However, the evidence for the effectiveness of rTMS on symptoms in veterans with SCZ is still low. Recent meta-analyzing data in the general population with SCZ demonstrated that compared with sham, rTMS administration has beneficial effects on auditory hallucinations and negative symptoms with low-medium average effect sizes (16–18). Conversely, other studies reported that real rTMS was not superior to sham treatment (19). Particularly, for the long-term hospitalized patients with SCZ, there is mixed evidence for the efficacy of rTMS on symptoms (20).

A special group of patients with SCZ are veterans in psychiatric hospitals. Most of the veterans with a mental disorder have experienced high levels of stress and have medical comorbidities and suicide risk (21). Most of the previous studies have assessed the efficacy of rTMS on the clinical outcomes in veterans with major depressive disorders (MDD) and posttraumatic stress disorder (PTSD) symptoms (22–24). Unlike in the general population, the evidence about the effectiveness of rTMS has been inconsistent and controversial among veterans (24, 25). Just a few studies have specifically explored the role of rTMS on clinical symptoms in veterans with SCZ.

As noted earlier, the dorsolateral prefrontal cortex (DLPFC) is associated with top-down impulsivity and inhibitory control (26, 27). The inhibitory control of DLPFC plays a critical role in executive function, visual-spatial working memory, and behaving in an appropriate manner, which are related to SCZ (28). Accumulating evidence from autopsy studies on presynaptic and postsynaptic elements supports the hypothesis that neuronal connectivity in the DLPFC of patients with SCZ is poor (29–31). Although abnormalities in the DLPFC are not the only pathological mechanism of SCZ, left DLPFC has been a target for rTMS in the treatment of SCZ in a majority of prior studies (32–35). At present, only one study regarding the effect of 20 Hz rTMS on clinical symptoms has been attempted in Chinese veterans with SCZ (36). They found that 20 Hz rTMS administration improved the excitement symptoms in SCZ. It is, therefore, warranted to further explore whether rTMS is a more effective intervention therapy in SCZ.

It is well known that rTMS is widely used in psychotic disorders to relieve negative symptoms and auditory hallucination and improve cognition functions, including SCZ (19, 36–40). Given the poor outcomes and difficulties of successful treatment, long-term hospitalized veterans with SCZ warrant the exploration of advanced treatment options beyond medications. Therefore, this study was designed to evaluate the effect of high-frequency (HF) rTMS on clinical symptoms in veterans with SCZ. We hypothesized that HF-rTMS over DLPFC for four consecutive weeks would significantly reduce the symptoms in veterans with SCZ.

## Methods

### Participants

A total of 47 long-term hospitalized veterans who have been diagnosed with SCZ according to the Diagnostic and Statistical Manual of Mental Disorders, Fifth Edition (DSM-5) were recruited in Hebei Province Veterans Hospital. The protocol was approved by the Institutional Review Board of the hospital. Written informed consent forms of all patients were signed. Inclusion criteria were (1) male, (2) Han Chinese population, (3) no modified electroconvulsive therapy (MECT) treatment in the past 6 months, (4) stable type of first- or second-generation antipsychotics and dose for more than 6 months, and (5) without abuse dependence except for smoking and alcohol. Exclusion criteria were (1) with risk of self-harm or suicide attempts, as assessed by the Nurses’ Global Assessment of Suicide Risk (NGASR); (2) any other mental illnesses diagnosed by an experienced psychiatrist; (3) a family history of epilepsy; (4) mental retardation; (5) pregnant or breastfeeding; and (6) with major physical diseases or brain disease including brain trauma, multiple sclerosis seizure, dementia, epilepsy, aneurysm, Huntington’s disease, brain tumor, stroke, and severe headache for unknown reasons. All veterans with SCZ have been hospitalized in the specific wards in our hospital. They are inpatients and therefore it was very convenient to use the rTMS treatment facilities.

**FIGURE 1:**
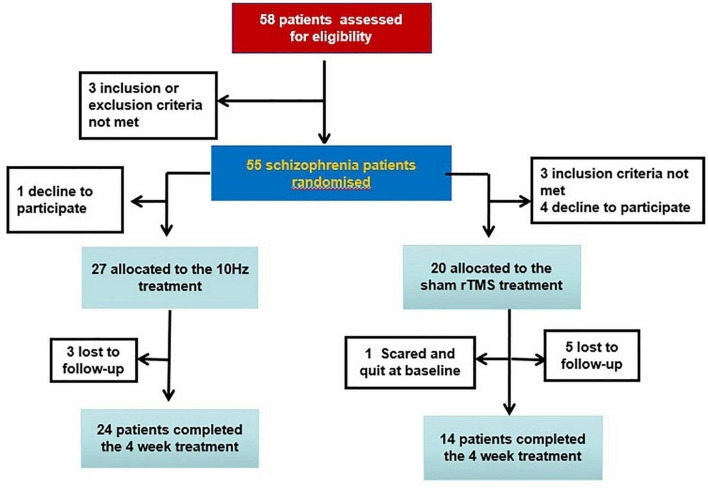
Flowchart.

Participants were randomly assigned to either the sham (*n* = 20) or real (*n* = 27) group. One patient in the sham group quit at baseline; two patients in the real group and five patients in the sham group were lost to follow-up at the end of the treatment (Figure 1). Antipsychotics consisted of olanzapine (*n* = 6), clozapine (*n* = 27), risperidone (*n* = 5), sulpiride (*n* = 1), chlorpromazine (*n* = 1), amisulpride (*n* = 1), haloperidol (*n* = 2), aripiprazole (*n* = 1), and quetiapine (*n* = 3). Switching the type of antipsychotic or changing the dose was not allowed in this study.

### Neuronavigated HF-rTMS treatment

This was a randomized, sham-controlled, and double-blind clinical trial. Random grouping is performed by a technician who did not participate in this study. First, a computer-generated randomization list was compiled by simple randomization. After enrollment, an independent third party randomly assigned patients to 10 Hz or the sham group according to the computer-generated random identification number they received. Researchers, scale raters, and patients were blinded to the random numbers. Only one rTMS operator in the Department of Physical Therapy who performed the rTMS treatment was aware of the assignment; however, he did not participate in the study.

Neuronavigated rTMS was administrated on the left DLPFC once a day, five times each week for four consecutive weeks with the MagStim Rapid2 Stimulator (23). The exact location of the left DLPFC was identical to our previous study (36), i.e., the superior region Brodmann area (BA) 46 and the posterior region BA9. All patients underwent a 0.3T MRI scan (BrivoMR235, Xinaobowei, Inc., China), and each patient’s MRI data were input into the Brainsight TMS Navigation system (Polaris Vicra Position Sensor, Northem Digital Inc., Canada). Finally, the Brainsight navigation system accurately located the TMS coil to the left DLPFC for each patient. rTMS Magstim’s 70 mm figure-of-8 was used in the real group. rTMS stimulations occurred at a power of 110% of MT for 3-s intervals with a 27-s inter-train interval (20 trains and total stimuli 24,000). In the sham group, the patients followed the same procedure as the real group except for a false coil, which looks identical to the real coil, replicates pulse noise, and mimics the sensation of real stimulation. Participants cannot distinguish whether they were assigned to real rTMS treatment or sham stimulation.

### Measurements

The primary outcome measure was the clinical symptoms. The symptoms were assessed at baseline and 4-week follow-up using the Positive and Negative Syndrome Scale (PANSS). The PANSS was evaluated by four experienced psychiatrists blinded to the randomization number. Before the start of this trial, to ensure consistency and reliability of PANSS rating across the trial, four raters simultaneously attended a training session. After training, the correlation coefficient of the PANSS total score by repeated evaluations was maintained at greater than 0.8.

In this study, the five-factor model of symptoms was applied to create phenotypic dimensions of SCZ (41), which consists of a positive factor (P1 + P3 + P5 + G9), negative factor (N1 + N2 + N3 + N4 + N6 + G7), an excitement factor (P4 + P7 + G8 + G14), a depressive factor (G2 + G3 + G6), and a disorganization factor (P2 + N5 + G11). In this way, it provides a broader dimension of the patient’s symptoms.

### Routine laboratory blood analysis

The peripheral blood of all participants was collected at 7 a.m. after 12 h of fasting. Routine biochemical markers including blood sugar, lipid profiles, hormone, and cell counts were measured in the medical laboratory using commercially available kits with an automatic biochemistry analyzer AU2700.

### Statistical analysis

Comparisons of demographic characteristics and clinical symptoms at baseline were performed by a chi-square analysis or ANOVA between real and sham groups. Intention-to-treat (ITT) analyses were performed for the patients who dropped out after the first week.

Our primary hypothesis was tested by the repeated-measures (RM) ANOVA to investigate the effect of 10 Hz rTMS on clinical symptoms. For the dependent variables, two time points (baseline and week 4) were a repeated measure within-effect, and grouping was a between-effect. If the interactive effect (group-by-time) was significant, a paired sample *t*-test was used to analyze within-group clinical symptoms. In addition, the difference between the real and sham groups at week 4 was compared by ANCOVA with the baseline scores as covariates. If the time-by-group interactive effects were not significant, no further statistical analyses were conducted. Pearson’s correlation analysis or Spearman’s correlation was performed to find whether the baseline biomedical parameters or the changes in those molecules were associated with the improvement of symptoms. A regression analysis was adopted to identify the association between improvement of symptoms and baseline values or changes in the biomedical markers.

Two-tailed significance values were used, and significance levels were set at 0.05.

## Results

### Demographic and clinical symptoms at baseline

Table 1 shows that there was no statistical difference in demographic data between the real and sham groups at baseline (all *p* > 0.05). Also, no significant difference in the PANSS total score or its three subscores was observed between the two groups (all *p* > 0.05). The baseline demographic data and clinical symptoms of the two groups were well matched.

**TABLE 1:** Demographic and clinical data of active and sham rTMS groups at baseline.

	Sham rTMS (*n* = 19)	10 Hz (*n* = 27)	*X*^2^ or *F(p)*
Age (yrs)	55.6 ± 10.3	52.7 ± 9.1	1.0 (0.32)
Education (yrs)	8.1 ± 2.1	8.4 ± 2.0	0.27 (0.60)
Age of onset (yrs)	21.2 ± 1.8	20.2 ± 1.6	3.4 (0.07)
Duration of illness (yrs)	34.5 ± 10.2	32.5 ± 8.8	0.5 (0.49)
Hospital time	6.9 ± 3.0	6.1 ± 2.9	0.8 (0.37)
Dose of antipsychotics	413.7 ± 235.9	410.5 ± 218.5	0.02 (0.98)
**Baseline PANSS scores**			
Total score	78.6 ± 24.2	76.7 ± 20.9	0.08 (0.77)
P subscale	14.3 ± 9.0	13.4 ± 5.6	0.2 (0.66)
N subscale	29.7 ± 9.1	29.0 ± 8.7	0.07 (0.80)
G subscale	34.6 ± 9.1	34.3 ± 9.7	0.01 (0.92)

PANSS, Positive and Negative Syndrome Scale; P, positive symptom; N, negative symptom; G, general psychopathology; RBANS, repeatable battery for the assessment of neuropsychological status; Dose daily antipsychotic dose (mg) chlorpromazine equivalent.

### Repetitive transcranial magnetic stimulation treatment on clinical symptom

The PANSS five-factors of patients with SCZ are shown in Table 2. RM ANOVA revealed a significant interaction effect on the positive factor (*F* = 4.3, *p* = 0.04) (Table 2). However, the main effect of time or group was not significant (all *p* > 0.05). Also, no significant interaction effect or main effect of time or group was observed on the negative factor, excitement factor, depressive factor, and disorganization factor (all *p* > 0.05).

**TABLE 2:** PANSS five-factor at baseline and at follow-up after treatment in real and sham rTMS groups.

	Baseline (*n* = 46)	Week 4 (*n* = 46)	Group effect *F(P)*	Time effect *F(P)*	Group × Time *F(P)*
**Positive factor**			0.1 (0.76)	1.2 (0.29)	4.3 (0.04)
Sham	9.0 ± 6.1	9.1 ± 6.2			
10 Hz	8.7 ± 5.2	8.3 ± 4.9			
**Negative factor**			0.04 (0.85)	4.2 (0.05)	0.02 (0.88)
Sham	25.1 ± 6.8	24.4 ± 6.1			
10 Hz	25.4 ± 6.7	24.8 ± 6.5			
**Disorganization factor**			0.2 (0.65)	0.1 (0.33)	0.03 (0.86)
Sham	9.3 ± 4.8	9.1 ± 4.8			
10 Hz	8.7 ± 3.5	8.5 ± 3.7			
**Excitement factor**			0.01 (0.92)	1.2 (0.28)	0.1 (0.74)
Sham	5.5 ± 2.9	5.6 ± 3.4			
10 Hz	5.3 ± 1.9	5.6 ± 2.3			
**Depressive factor**			0.03 (0.86)	3.4 (0.07)	0.4 (0.51)
Sham	4.1 ± 1.4	3.9 ± 1.0			
10 Hz	4.1 ± 1.4	3.9 ± 1.0			
**Positive subscale**			0.3 (0.61)	0.4 (0.51)	0.9 (0.35)
Sham	14.3 ± 9.0	14.4 ± 9.4			
10 Hz	13.4 ± 5.6	13.1 ± 5.3			
**Negative subscale**			0.07 (0.79)	4.2 (0.05)	0.002 (0.96)
Sham	29.7 ± 9.1	28.9 ± 8.0			
10 Hz	29.0 ± 8.7	28.3 ± 8.5			
**General psychopathology subscale**			0.01 (0.94)	3.2 (0.08)	0.02 (0.88)
Sham	34.6 ± 9.1	33.6 ± 8.7			
10 Hz	34.3 ± 9.7	33.4 ± 8.7			
**PANSS total score**			0.1 (0.76)	5.3 (0.026)	0.01 (0.92)
Sham	78.6 ± 24.2	76.9 ± 23.8			
10 Hz	76.7 ± 20.9	74.8 ± 19.7			

Further analyses in the two groups revealed that compared with baseline, a significant decrease was found in the scores of positive factors after treatment with 10 Hz rTMS in the real group (*Z* = −2.1, *p* = 0.038), but there was no change in the sham group (*p* > 0.05). Improvement in positive factor in the real rTMS group was clinically meaningful for veterans with SCZ, with a 0.4 (0.1)-point reduction that reflected a low effect size (Cohen’s *d* = 0.08).

### Repetitive transcranial magnetic stimulation treatment on blood biomedical molecules

We then analyzed whether the improvement of symptoms was associated with the changes from baseline values to follow-up values in the biomedical molecules. The results showed that there was no significant association between symptoms improvement and changes in the white blood cells (WBC) and red blood cells counts or the levels of blood fasting glucose, prolactin, triglycerides, total cholesterol, low-density lipoprotein (LDL), high-density lipoprotein (HDL), apolipoprotein A (Apo A), apolipoprotein B (Apo B), C-reactive protein (CRP), homocysteine, testosterone, thyroid-stimulating hormone (TSH), free thyroxine (fT4), total thyroxine (tT4), free triiodothyronine (fT3) and total triiodothyronine (tT3), glutamic pyruvic transaminase (ALT), and glutamic oxaloacetic transaminase (AST) (all *p* > 0.05). However, we found that the WBC count at baseline was negatively associated with the improvement of positive factor (*r* = −0.42, *p* = 0.029), which was further confirmed by the regression analysis (β = −0.40, *t* = −2.25, *p* = 0.034).

### Side effects

No rTMS-related serious side effects were observed in this study; two patients (one in the real group and one in the sham group) felt dizzy with the procedure; one patient in the sham group complained of scalp pain, and seven patients reported mild discomfort during rTMS treatment.

## Discussion

To the best of our knowledge, this study provides the first evidence for 10 Hz rTMS over the left DLPFC on clinical symptoms in long-term hospitalized veterans with SCZ. We found that HF-rTMS treatment for 4 weeks produced an effective therapeutic benefit for positive symptoms in veterans with SCZ. In addition, 10 Hz rTMS administration was safe with few side effects (including scalp pain, dizziness, and mild discomfort) consistent with those known safety characteristics of rTMS.

Our findings provide further evidence for a potential treatment add-on of HF-rTMS for clinical symptoms in veterans. It is worth noting that our cohort is made up of a special population, who are presumed to have experienced great stress more than 30 years ago. The patients are veterans with a mean illness duration of 33.3 (9.3) years, the average age is 54.0 (9.5) years, and the average onset age is 20.7 (1.7) years. According to the military service law in China, the minimum age for voluntary recruitment to enlist in military branches is 18 years. It is concluded that the majority of patients in this study were diagnosed with SCZ during the first 3 years after enrollment. Our findings showed that HF-rTMS was still effective for special populations such as long-term hospitalized veterans with SCZ. However, our findings were inconsistent with a previous controlled trial in veterans which found that real 20 Hz rTMS for 8 consecutive weeks showed a marginally significant difference in the clinical outcomes of veterans with SCZ compared with sham (36). All participants in both studies were long-term hospitalized veterans, so they were administrated rTMS regularly with the monitoring of the nurses and researchers. We speculated that the difference in outcomes between the two studies was due to the different stimulation parameters, such as frequency (10 vs. 20 Hz) or duration (4 vs. 8 weeks), which account for the discrepancy.

Our findings indicate the importance of delivering rTMS for long-term hospitalized veterans with SCZ. The exact mechanisms of the efficacy of rTMS treatment for positive symptoms in veterans with SCZ are still unknown. It is known that the prefrontal cortex is involved in the regulation of behavior such as executive function and inhibitory control (42). rTMS targeting the prefrontal cortex reduces the positive symptoms via the ability to alter the cortical activity of local and remote sites of stimulation. Prior studies have shown that HF-rTMS over the DLPFC potentially inhibits the hypoactivity in the DLPFC and related temporal lobe to reduce positive symptoms (43). Studies have also investigated the therapeutic effects of rTMS over DLPFC in positive symptoms of patients with SCZ. For example, a randomized controlled study using rTMS to the left DLPFC in patients with treatment-resistant SCZ showed significant reductions in the Brief Psychiatric Rating Scale (BPRS) scores compared with sham after treatment (44). However, this is only our speculation, and the evidence remains limited since there were not many studies in this field, especially in veterans with SCZ. Therefore, the precise cause and mechanism behind the symptom improvement warrant to be further investigated in a large cohort of SCZ veterans.

We found that age, onset age, body mass index, levels of blood sugar, blood lipid, endocrine, and metabolic parameters at baseline were not associated with the improvement of positive symptoms. In addition, rTMS treatment did not change the levels of these biomedical markers, and the symptom improvements were also not associated with the changes in the biomedical markers. Interestingly, we found that the WBC count at baseline was a predictor of symptom improvement after treatment with rTMS in veterans. The positive symptoms of veterans with more WBC counts at baseline were more difficult to improve. Increasingly advances have identified the correlation between the risk of SCZ and abnormal immune system function, such as the immune cell numbers and inflammatory markers (45, 46). We speculate that those veterans with more WBC counts may have a chronic or acute inflammatory response since it is well known that whole white cells are the hallmark cell of the immune system and WBC counts are predictive of a marker of inflammation in clinical practice (47, 48). Therefore, those patients with low WBC counts may have a low inflammatory response, which may lead to better improvement of positive symptoms.

However, there is a lack of negative symptom improvements in this study, which is contrary to our expectations. Notably, as we described above, previous evidence has supported the efficacy of HF-rTMS on the negative symptom dimension when the left DLPFC is stimulated (16–18). We speculated that this discrepancy may be due to bias in patients (veterans vs. general patients) recruited among studies. Additionally, another difference is that the current standard treatment protocols foresee stimulation on the temporoparietal junction to treat negative symptoms in patients with SCZ (49, 50), whereas the veterans in this study were stimulated on the left DLPFC to treat negative symptoms. Additionally, we only found that patients allocated to active treatment showed improvements at the PANSS positive factor of the five-factor model but not at the PANSS positive subscale. Although a significant association was observed between excitement factor and positive factor at baseline, there were no significant associations between improvements in excitement factor or other factors and positive factor. A possible explanation for these findings was that the sample size of the groups was small and the duration of rTMS treatment was relatively short. In addition, patients recruited in this study were long-term hospitalized veterans. Therefore, there were no statistically significant improvements in clinical symptoms, except for the positive factors.

Limitations of this study include the relatively small sample size. Thus, the negative results of the lack of beneficial effects of rTMS on other PANSS symptoms in veterans with SCZ reported in this study may be due to the low statistical power of the small size of samples. Moreover, the subgroup analysis was also limited because of the small sample size. The second limitation is that all participants in this study are male veterans diagnosed with SCZ. Thus, our results cannot be generalized to female veterans or the general population, which limited the generalization of main findings in clinical applications. The third limitation is that we did not continue to evaluate the symptoms after the completion of rTMS treatment. Thus, less is known about whether there is a long-lasting beneficial effect of HF-rTMS on positive symptoms in this study. Fourth, in this study, abuse dependence was not assessed by urine analysis or other methods (e.g., DSM-5 criteria), but only by self-reported drug use. Fifth, pre- and post-treatment neuroimaging techniques to assess any neural changes were not included in this study. Sixth, we measured the severity of clinical symptoms using only the PANSS in patients and did not use other scales to assess the symptom severity, such as the Clinical Global Impression (CGI). Seventh, since 0.3T MRI has a very slow spatial resolution, especially for neuroimaging study purposes, this is a limitation for this study, which could additionally explain the lack of clear effects at the clinical and symptomatological level.

Taken together, our preliminary study investigating the efficacy of neuronavigated HF-rTMS stimulation on clinical outcomes shows that rTMS stimulation is beneficial to the positive symptoms in veterans with SCZ. Our findings provide further evidence that rTMS is a reasonable treatment option to relieve the clinical symptoms and has great clinical significance. Additional studies are warranted to confirm the therapeutic effectiveness of positive symptoms in long-term hospitalized veterans with SCZ using large sample size and inclusion of different ethnic groups.

## Data availability statement

The raw data supporting the conclusions of this article will be made available by the authors, without undue reservation.

## Ethics statement

The studies involving human participants were reviewed and approved by the Institutional Review Board of Hebei Province Veterans Hospital. The patients/participants provided their written informed consent to participate in this study.

## Author contributions

XW, MX, and XS were responsible for the study design, statistical analysis, and manuscript preparation. XL, HY, LZ, YC, and YS were responsible for recruiting the patients, performing the clinical rating, and collecting the clinical data. XS, MX, and HY were involved in evolving the ideas and editing the manuscript. LL and MX were involved in writing the protocol and co-wrote the manuscript. All authors have contributed and approved the final manuscript.
